# Effects of sex but not race and geographic origin on vaccine-induced HIV-specific antibody responses

**DOI:** 10.3389/fimmu.2025.1601865

**Published:** 2025-09-23

**Authors:** Wilbert Mbuya, Augusta Horvath, Kathrin Held, Lucas Maganga, Michael Hoelscher, Linda-Gail Bekker, Ann Duerr, Zoe Moodie, Gavin Churchyard, Michael C. Keefer, Edna Viegas, Christiane Moog, Christof Geldmacher, Mkunde Chachage

**Affiliations:** ^1^ National Institute for Medical Research-Mbeya Medical Research Center (NIMR-MMRC), Mbeya, Tanzania; ^2^ Institute of Infectious Diseases and Tropical Medicine, LMU University Hospital, LMU Munich, Munich, Germany; ^3^ German Center for Infection Research (DZIF), Partner Site Munich, Munich, Germany; ^4^ Fraunhofer Institute for Translational Medicine and Pharmacology ITMP, Immunology, Infection and Pandemic Research, Munich, Germany; ^5^ Unit Global Health, Helmholtz Zentrum München, German Research Centre for Environmental Health (HMGU), Neuherberg, Germany; ^6^ Desmond Tutu HIV Foundation, University of Cape Town, Cape Town, South Africa; ^7^ Vaccine and Infectious Disease Division, Fred Hutchinson Cancer Center, Seattle, WA, United States; ^8^ Aurum Institute, Parktown, South Africa; ^9^ School of Public Health, University of Witwatersrand, Johannesburg, South Africa; ^10^ Department of Medicine, Vanderbilt University, Nashville, TN, United States; ^11^ Department of Medicine, University of Rochester School of Medicine and Dentistry, Rochester, NY, United States; ^12^ Instituto Nacional de Saúde (INS), Maputo, Mozambique; ^13^ UMR_S 1109 INSERM, Fédération de Médecine Translationnelle de Strasbourg (FMTS), Université de Strasbourg, Strasbourg, France; ^14^ Vaccine Research Institute (VRI), Créteil, France; ^15^ Department of Microbiology and Immunology, University of Dar es Salaam-Mbeya College of Health and Allied Sciences (UDSM-MCHAS), Mbeya, Tanzania

**Keywords:** HIV-1, vaccines, race, sex, antibody, HVTN 204

## Abstract

**Background:**

Sex, race and geographic location may affect vaccine-induced immune responses, yet few preventive HIV vaccine trials have systematically evaluated such effects. The main objective of this study was therefore to examine the role of these factors on vaccine-induced HIV-specific immune responses within the HVTN 204 trial. This randomized, double-blinded, placebo-controlled phase 2 trial enrolled 480 Black and Caucasian adults from Africa and the Americas, who received a trivalent DNA-HIV-1 vaccine prime followed by a rAd5 vector HIV-1 vaccine boost.

**Methods:**

Available serum samples from baseline and four weeks after the final vaccination boost from Black (n=85, 59% female) and Caucasian (n=49, 51% female) HVTN 204 vaccine recipients from South Africa and the United States of America were studied using an Enzyme-Linked Immunosorbent Assay to determine titers of Envelope-specific IgG1, IgG3 and IgA antibodies. Recognition of linear Envelope peptide-specific IgG responses was mapped in a randomly selected subgroup analysis using a custom-designed peptide microarray (n = 41, 49% female). Associations between vaccine-induced Envelope-specific antibody responses and sex assigned at birth (female or male), race and geographic location were then analyzed by the Mann-Whitney U test, Fisher’s exact test and multivariate logistic regression.

**Results:**

Four weeks post-final vaccination boost, we observed that Envelope-specific antibody titers were significantly increased for IgG1 but reduced for IgA in females (female vs. male median titer: 900 vs. 300, p=0.030 and <100 vs. 100, p=0.007, respectively). Multivariate logistic regression confirmed that female sex increased the odds for higher Envelope-specific IgG1 and low IgA titers compared to males. In terms of antibody epitopes, the V2 region was more frequently recognized in females than males (p=0.008). Race and geographic location had no apparent influence on antibody isotype titers investigated.

**Conclusion:**

Female sex was associated with higher vaccine-induced IgG Envelope-specific binding antibody titers and recognition of V2 region of HIV Envelope in HVTN 204 volunteers. No such associations were detected for race or geographic location. Understanding biological factors driving these sex-based differences may improve the design of a new generation of HIV vaccine candidates.

## Introduction

Host genetics, sex and geographic location can influence immune responses to vaccines, such as measles, influenza and yellow fever (1–3). Some studies have suggested that Africans when compared to Caucasians have reduced HIV vaccine-induced immune responses after vaccination with HIV Envelope (Env) encoded in Adenoviral vectors (4, 5). African vaccinees also showed higher but less potent titers of HIV-neutralizing antibodies (Nab) against primary HIV-1 isolates compared to Caucasians, indicating that geographic location and/or race of vaccinees may influence vaccine-induced adaptive immune responses (4). Of note, attempts to replicate RV144’s moderate HIV vaccine efficiency observed in the Thai volunteers in a South African (SA) high-risk cohort were unsuccessful (6). Similarly, the recent “PrEPVacc” trial, which assessed the efficacy of two experimental HIV vaccine regimens in African populations was stopped after failing to demonstrate efficacy in preventing HIV acquisition (7). HVTN 204 was a large multi-country, transcontinental, placebo-controlled phase 2 HIV vaccine trial conducted from 2008 to 2010 in the United States of America (US) and South Africa (SA), enrolling Black and Caucasian volunteers (8). The vaccinees received a multiclade VRC-HIV DNA, followed by administration of a recombinant Adenoviral vectored vaccine rAd5-HIV. Vaccination was safe and induced T-cell and a high frequency of antibody responses to the vaccine-encoded HIV-Env and Gag antigens in 70% and over 80% of vaccinated volunteers, respectively (8, 9). While a similar vaccine in a follow-up study did not reduce the rate of HIV-1 acquisition in high-risk US volunteers (9), the HVTN 204 study is optimally suited to address whether sex, geographic location and/or race can influence the immunoglobolin (Ig) isotype, epitope recognition patterns and/or magnitude of HIV vaccine-induced antibody responses.

Of note, IgG recognition of the V1V2 loop region of the HIV Env correlated with RV144 vaccine efficacy among Thai volunteers (10–14), while binding of plasma IgA antibodies to Env correlated directly with the rate of HIV-1 infection in RV144 vaccinees (13). A plausible explanation is that while IgA is the most abundant antibody in mucosal sites, it can inhibit IgG mediated effector functions (15, 16). Differences in V1V2-specific IgG responses and IgA titers after HIV-1 vaccination between African and non-African vaccine recipients could therefore also hypothetically contribute to the failure of multiple recent phase 2b/3 vaccine trials in African populations.

Recently, we reported higher total immunoglobulin levels but similar IgG and IgA vaccine-induced responses to HIV-1 Env antigens in Black South Africans compared to US Caucasians in HVTN 204 trial participants after vaccination (17). Therefore, we now examined the influence of race, sex and geographic location on the frequency, linear epitope specificity and isotypes of Env-specific antibody following DNA/rAd5 prime-boost vaccination during HVTN 204.

## Methods

### Study design and population

This study used a subset of serum samples from HVTN 204 – a randomized, double-blinded, placebo-controlled phase 2 clinical trial conducted from 2008 to 2010, which evaluated the safety and immunogenicity of a multiclade HIV-1 DNA vaccine, followed by a multiclade recombinant Adenoviral Vector HIV-1 vaccine boost in adults. HVTN 204 enrolled 480 male and female participants without HIV-1 from the Americas [US, Haiti, Jamaica, and Brazil] and SA (Clinicaltrials.gov: NCT00125970) (8), making HVTN 204 amongst the earliest phase 2 HIV-1 vaccine trials that enrolled participants across different races and geographic locations. The male or female sex of participants was recorded as “sex assigned at birth”. Trial participants received either three multivalent DNA-HIV prime immunizations followed by a single rAd5-HIV boost immunization (n=240), or a placebo (n=240). Among vaccine recipients, 210 were from the US (n=90) and SA (n=120). This sub-study focused on analyzing available serum samples (n=134) from Black or Caucasian vaccinees in the US and SA that were collected in HVTN 204 at baseline and four weeks after the final vaccine boost (**Supplementary Figure S1**).

### Ethics approval

Participants in HVTN 204 provided written informed consent for future use of their data and specimens. Approval for our use of the data and specimens was obtained from relevant ethics committees. Protocol approval details are as published by Churchyard et al. (8).

### Binding enzyme-linked immunosorbent assays

IgG1-, IgG3- and IgA-specific binding ELISA responses to a recombinant HIV-1 subtype C Envelope protein-CN54rgp140 were measured in serum samples at baseline, and at four weeks post-final vaccination boost, in a 3-fold (for IgG isotypes) or 5-fold (for IgA) dilution series as previously described by Joachim et al. (18). Absorbance (optical density = OD) was read immediately at 450 nm using an ELISA plate reader (Tecan, San Jose, US). A sample was considered positive for anti-CN54rgp140 IgG isotypes at 1: 100 or 1: 300 dilutions if the mean OD value was more than twice that of the pre-immunization sample at 1:100 or 1:300 dilutions, respectively. A sample was considered positive in a dilution >1:300 if the absorbance value was more than twice the mean of the pre-immunization sample run at a 1:300 dilution. A positive vaccine-induced IgA response was defined as mean OD at 1: 100 or 1: 500 dilutions being more than twice that of the pre-immunization sample at 1:100 or 1:500 dilutions, respectively. A sample was also considered as positive for anti-CN54rgp140 IgA at a dilution >1:500 if the absorbance value was more than twice the mean of the pre-immunization sample run at a 1:500 dilution. Data were reported as reciprocal end-point titers (end-point titer being the last dilution at which a sample shows a positive response).

### Peptide array

A peptide microarray (JPT, Berlin) approach was used to assess IgG binding to the HIV-1 Env peptides as described previously by Horvath et al. (19). In brief, our custom microarray included 1034 overlapping linear 15-mer peptides to map the gp160 extracellular domain of the HIV-1 Env protein. The array backbone consists of 10 full-length Env immunogen sequences. Additionally, 15 previously identified immunodominant regions were covered by additional peptide variants of all HIV-1 clades covering the current epidemic. We measured serum samples from baseline and four weeks after final vaccination at a dilution of 1:100. Fluorescence signals were measured with a GenepixPro Scanner, 6.0 software (Molecular Devices, San José, CA, US) and converted into numeric intensity values with a maximum of 60000. Each peptide was assigned the correct position on the gp160 scaffold with the corresponding fluorescence intensity (FI) value. For the assignment to the gp160 alignment, we used a fasta file containing both the sequences of the array and the immunogen sequences of the HVTN 204 trial. For further analysis of peptide array IgG binding data, baseline values were subtracted from post-vaccination values using R studio (version 1.4.1106) and Microsoft Excel. To exclude background signals, we used a cut-off for positive signals of 2500 FI. Mean FI values per study group were calculated if at least 25% of the vaccinees showed a positive binding response against the respective peptide. Immunodominant regions (IDR) were defined by frequent (FOR of > 50%) and strong (mean FI > 10000) recognition of peptides in at least one of the four groups (US female, US male, SA female, SA male). If more than one overlapping peptide was recognized, the epitope with the higher response was selected for statistical analysis.

### Statistical analysis

Stata version 17 (StataCorp, US), Microsoft Excel (Microsoft, US), R version 4.x (R Core Team, 2024) and GraphPad Prism version 9 (GraphPad Software Inc, US) were used for statistical analysis. Two-tailed Mann-Whitney U test was done to compare titers or peptide recognition across participants’ sex, geographic location and race. The median age between females and males was also analyzed by Mann-Whitney U test. Fisher’s exact test was used in the comparison of all categorical analyses. Logistic regression was done to investigate the association between sex, age, and race with the frequency of positive antibody responses. Model fitness was assessed by Hosmer–Lemeshow Test whereby a model was considered a good fit if the p-value was greater than 0.05 for all models.

## Results

### Description of the cohort

A summary of the geographic location, age, sex, and race of study participants is provided in **Table 1**. Serum samples from 134 age- and sex-matched vaccinated participants with a median age of 26 years were analyzed. Over half (56%) were female, nonetheless, males and females had similar demographic characteristics (**Table 1**).

**Table 1:** Baseline demographic characteristics (n=134).

Variable		All (134) [n, (column %)]	Female (75) [n, (column %)]	Male (59) [n, (column %)]	P-value
Country	US	61 (45.5%)	31 (41.3%)	30 (50.9%)	0.298^a^
	South Africa	73(54.5%)	44 (58.7%)	29 (49.1%)	
Age, median (IQR)		26 (22-33)	26 (22-31)	25 (22-36)	0.554^b^
Race	Black	85 (63.4%)	50 (66.7%)	35 (59.3%)	0.470^a^
	Caucasian	49 (36.6%)	25 (33.3%)	24 (40.7%)	

Statistical tests: ^a^Fisher’s exact test, ^b^Mann-Whitney U test.

### Females and Black Africans have increased IgG1 background signals

**Figure 1:**
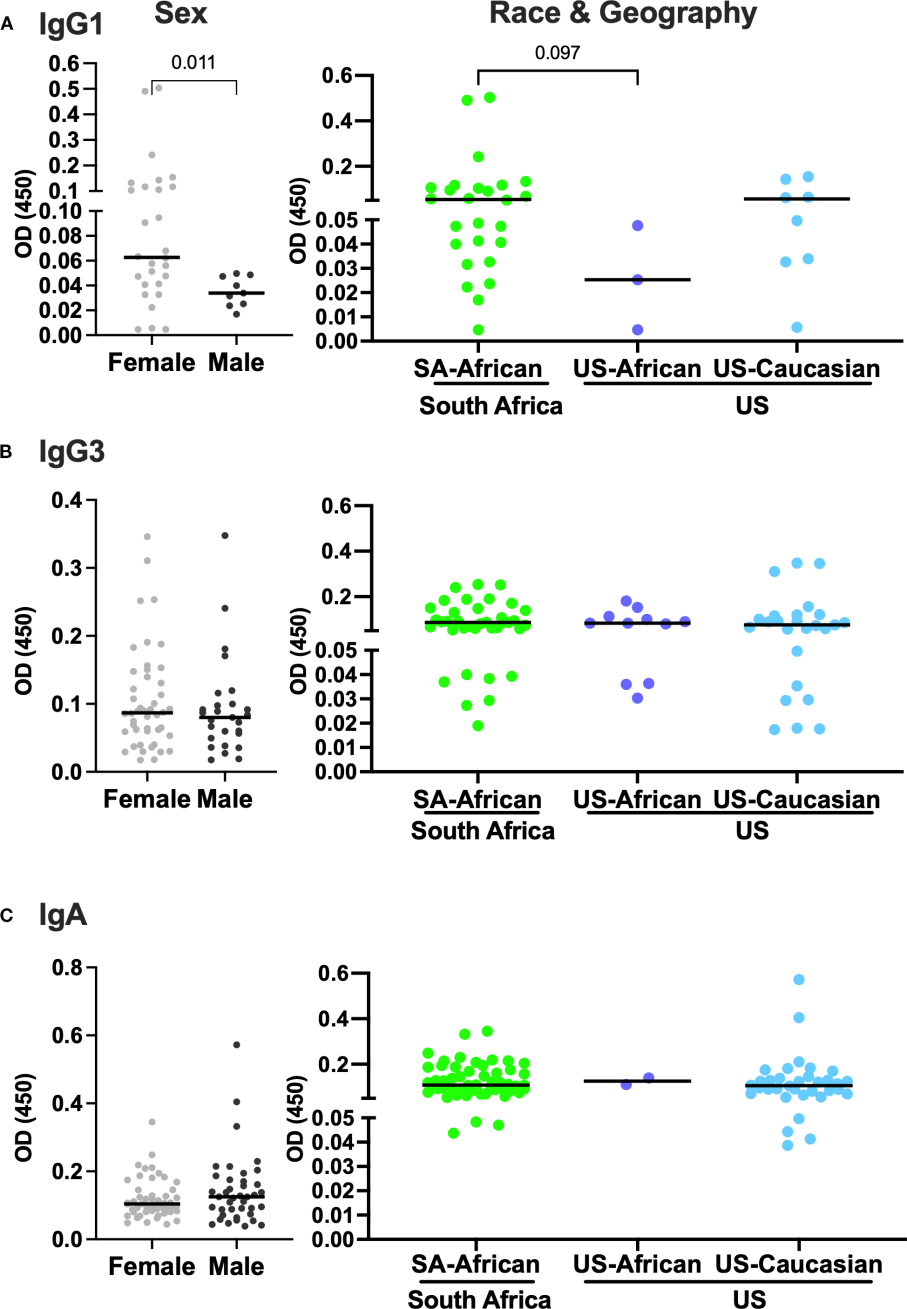
Females and South Africans have increased anti-CN54rgp140-specific IgG1 background signal. Optical densities (OD) in participants with background signal pre-vaccination are stratified by sex (left panels), race and geographic location (right panels) of participants. **(A)** IgG1 (n=37), **(B)** IgG3 (n=73), and **(C)** IgA (n=90). Each dot represents one individual. Median lines are indicated in graphs. Statistical analysis was performed using the Mann-Whitney U-test.

We first analyzed the background levels (at 1:100 dilution) of our anti-CN54rgp140 IgG and IgA antibody testing approaches at baseline, prior to the first vaccination in HVTN 204. Regardless of sex, race or geographical location and despite low pre-vaccination ODs, 32% (37/115), 54% (73/134) and 86% (90/105) of all pre-vaccinated individuals had 2-fold or higher ODs when compared to blank ODs for IgG1, IgG3 and IgA, respectively. Upon stratification by sex, females had a 1.8 times higher median IgG1 background signal compared to males (median OD: 0.062 vs 0.034, p = 0.011, **Figure 1**, left panel). Furthermore, Black South Africans had comparable pre-vaccination anti-CN54rgp140 binding IgG1 OD values when compared to Caucasians but slightly increased when compared to Black Americans (median IgG1 OD: 0.053 *vs* 0.025, p = 0.097, **Figure 1**, right panel). In South Africa, Black females had significantly higher background signals of anti-CN54rgp140 IgG1 compared to Black males (p = 0.011, **Supplementary Figure S2**).

### Sex influences isotype profile of vaccine-induced antibody responses

**Figure 2:**
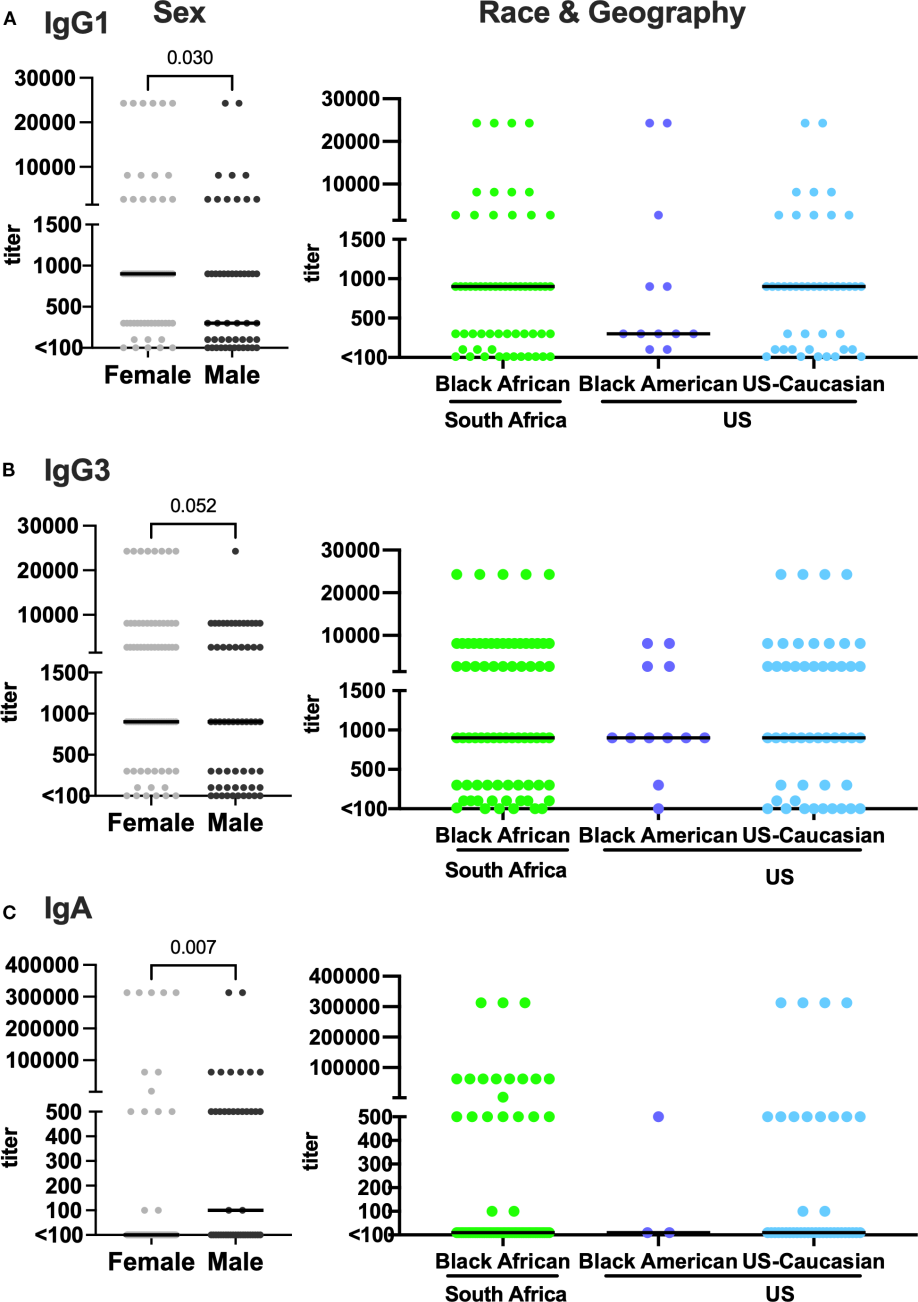
Increased IgG1 titers but decreased IgA titers in females. Titers in vaccinated participants are stratified by sex (left panels), race and geographic location (right panels) of participants. **(A)** IgG1 (n=115), **(B)** IgG3 (n=134), and **(C)** IgA (n=105). Each dot represents one individual. Median lines are indicated in graphs. Statistical analysis was performed using the Mann-Whitney U-test.

Next, we analyzed IgG (IgG1 and IgG3) and IgA titers to subtype C CN54rgp140 at 4 weeks post-final vaccine boost. While vaccine-induced anti-CN54rgp140 binding IgG1 and IgG3 antibodies were present in most participants (IgG1: 85.2% and IgG3: 88.0%, **Table 2**), IgA antibodies were detected in only 34% of vaccinees. A significantly higher proportion of female participants showed anti-CN54rgp140 IgG1 responses than males (92.3% *vs* 76.0%, p=0.018, **Table 2**). On the contrary, a low proportion of females mounted detectable anti-CN54rgp140 IgA responses compared to males (22.6% *vs* 51.2%, p=0.003, **Table 2**). Consistent with these findings, we also observed significantly higher anti-CN54rgp140 IgG1 and lower anti-CN54rgp140 IgA titers in females compared to males (anti-CN54rgp140 IgG1 median titers: 900 (IQR:300-1800) *vs* 300 (IQR:75-900, p=0.030); anti-CN54rgp140 IgA median titers: <100 (IQR: <100 -<100) *vs* 100 (IQR: <100 -500), p=0.007, **Figure 2**, left panels).

**Table 2:** Frequency of antibody responses to subtype C CN54rgp140 per isotype at four weeks post-final vaccination boost.

Antibody Isotype	All [n, (%)]	Female [n, (%)]	Male [n, (%)]	P-value*
IgG1	98/115 (85.2%)	60/65 (92.3%)	38/50 (76.0%)	**0.018**
IgG3	118/134 (88.0%)	69/75 (92.0%)	49/59 (83.0%)	0.178
IgA	36/105 (34.3%)	14/62 (22.6%)	22/43 (51.2%)	**0.003**

*Fisher’s exact test. Bold values indicate statistical significance (p<0.05).

When IgG1, IgG3 and IgA titers were compared in vaccinated individuals stratified by race and geographic location, we observed similar median titers for all antibody sub-classes across all groups (**Figure 2**, right panels). When further stratified by sex and geographic location, significantly higher anti-CN54rgp140 IgA titers were observed in South African males than in South African females (median: 100 *vs <*100, p = 0.042, **Supplementary Figure S3**).

### HIV-1 Env epitope recognition varies by geographic location and sex

**Figure 3:**
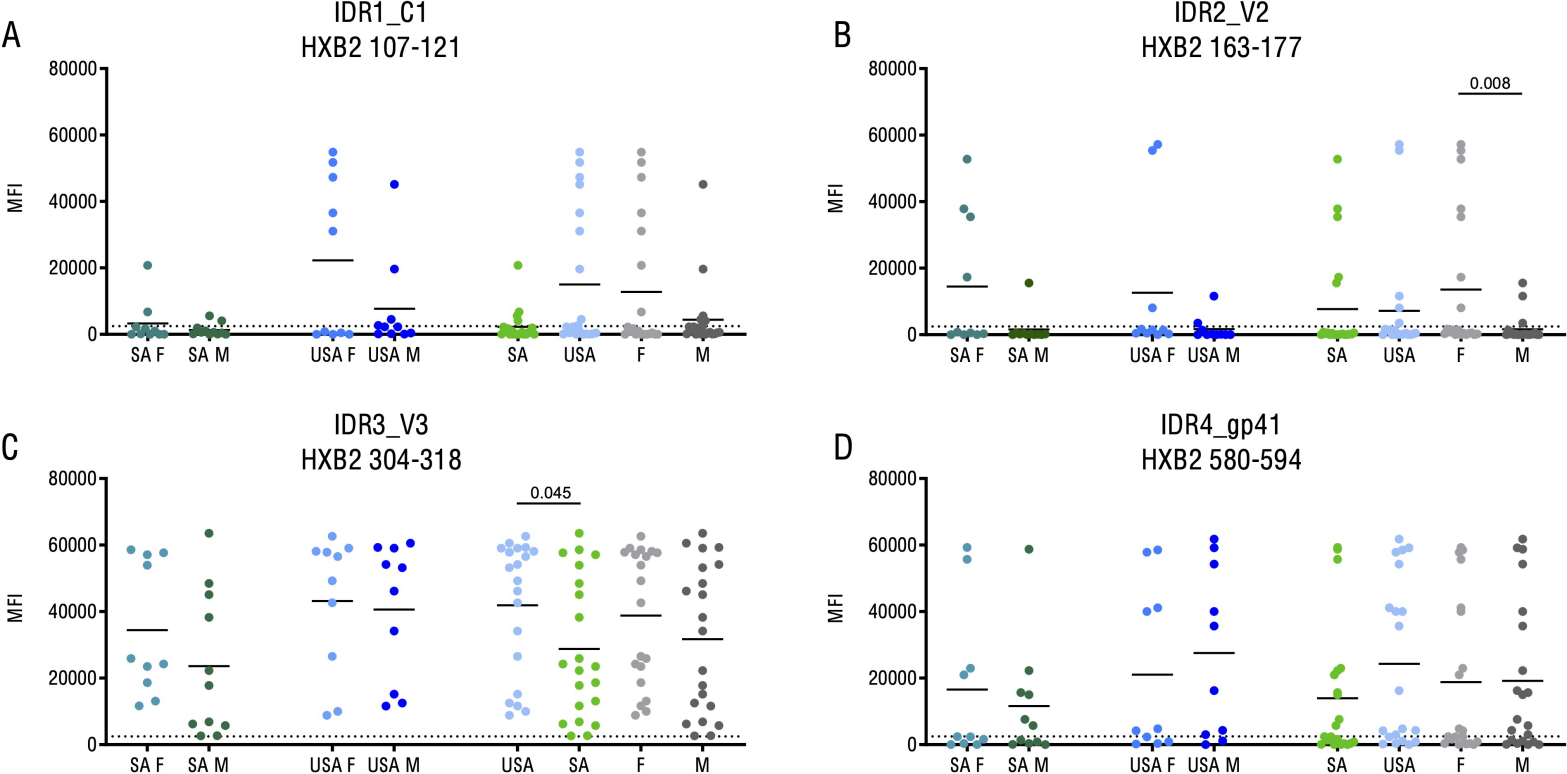
Variations in B cell epitope recognition based on both sex and geographic location. Comparison of IgG responses targeting single peptide immuno-dominant regions as measured by peptide microarray. Each graph **(A–D)** depicts mean FI values post-vaccination without baseline-correction for one representative peptide of the 4 immuno-dominant regions. Corresponding HXB2 amino acid positions are stated. Each symbol indicates the maximum FI value of one single study participant. The cut-off for positive signals is indicated by a dotted line. P-values were calculated using a Mann-Whitey-U test. Only p-values <0.05 are shown. n US F=10; n US M=10; n SA F=10M; n SA M=11. IDR, immuno-dominant region; F, female; M, male; US, United States of America; SA, Republic of South Africa.

To determine IgG antibody epitope specificity within the HIV-1 Env, peptide array analysis was conducted on a subgroup of 21 Black South Africans and 20 US Caucasian participants with equal proportions of males and females (**Supplementary Table S1**). Four prominent IDRs, as defined by high frequency of recognition and signal intensity, were detected after final vaccination boost: one within C1 (IDR1_C1; HxB2 gp160 pos 107-121; DIISLWDQSLKPCVK), another in V2 (IDR2_V2; HxB2 gp160 pos 163-177; TSIRGKVQKEYAFFY), followed by V3 (IDR3_V3; HxB2 gp160 pos 304-318; RKRIRIQRGPGRAFV), and finally, gp41 (IDR4_gp41; HxB2 gp160 pos 580-594; ILAVERYLKDQQLLG) (**Supplementary Figure S4**). While IDR3_V3 was the most immunodominant among these, IDR1_C1 exhibited higher frequency and intensity of recognition among participants from the US (response in 9/20; mean FI 15,008) than from South Africa (response in 4/21; mean FI 2,243) (p = 0.077; **Figure 3A**). Recognition of IDR1_C1 was slightly stronger in females (response in 7/20; mean FI 12,770) than males (response in 6/21; mean FI 4,374). Significant sex-specific differences were detected for IDR2_V2-specific responses, a region associated with protective immunity in RV144 (13), which demonstrated more frequent recognition in females (response in 7/20; mean FI 13,568) compared to males (response in 3/21; mean FI 1,629; p = 0.008; **Figure 3B**) across both the US and South African cohorts. Additionally, IDR3_V3 exhibited slightly stronger recognition among participants from the US (mean FI 41,874) than from South Africa (mean FI 28,760; p = 0.045; **Figure 3C**). Interestingly, IDR3_V3 – an epitope usually recognized with high frequency and intensity - was poorly recognized only in South African males (mean FI 23,600). Recognition of a linear epitope covering the start of the immunodominant region within gp41 (IDR4_gp41) did not show significant variation across the HVTN 204 participants (**Figure 3D**).

### Sex but not age, geographic location or race affects the frequency of HIV vaccine antibody responses

**Figure 4:**
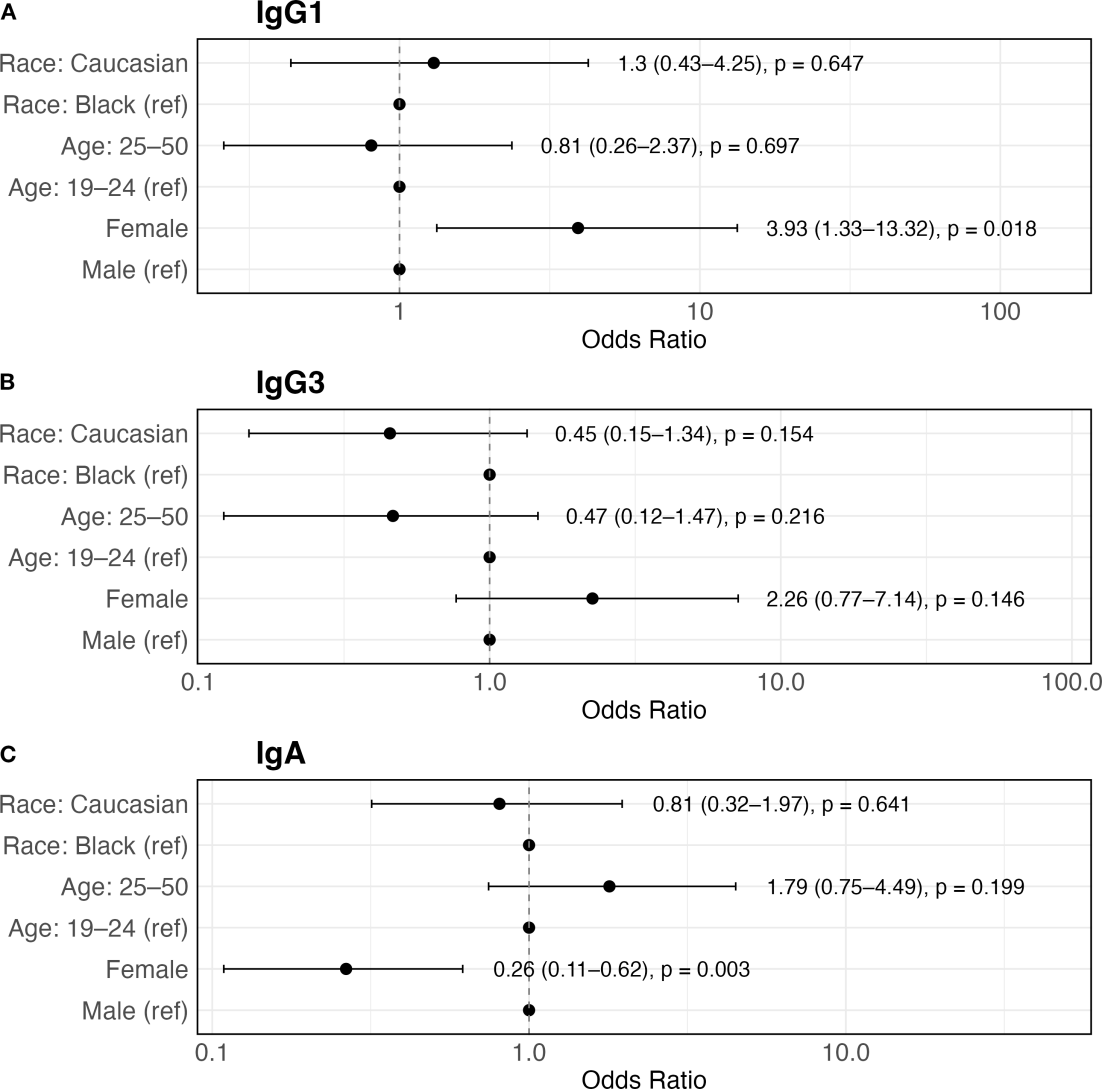
Forest plot displaying odds ratios for a positive antibody response influenced by sex, age and race. **(A)** IgG1 (n=115), **(B)** IgG3 (n=134), and **(C)** IgA (n=105). Forest plot displaying odds ratios for the interaction of positive IgG1, IgG3 and IgA antibody responses with sex, age (19–24 vs. 25–50 years) and race (Black vs. Caucasian) for HVTN 204 vaccinees. The individual risk factors are indicated on the y-axis; adjusted odds ratios and 95% confidence intervals (CI) are shown on the x-axis. The actual odds ratios and 95% CI are shown on the right side of each graph. Age stratification is adopted from the WHO classification of youth vs adult. Statistical analysis was performed by multivariate logistic regression.

Multivariate logistic regression was performed to evaluate the effects of sex, age, and race on the frequency of detectable antibody responses per isotype (**Figure 4**). For IgG1, females had 3.9 increased odds of a positive antibody response compared to males (95% CI: 1.33 – 13.32, p = 0.018). Interestingly, the opposite was observed for IgA where females had 74% lower odds for positive antibody response (95% CI: 0.11 – 0.62, p = 0.003). Of note, IgG3 was not affected by any of the aforementioned factors. Age and race had no statistically significant association with the frequency of antibody immune response for any of the analyzed immunoglobins.

## Discussion

Differences in Env-V1V2-specific IgG responses and Env-specific IgA titers after HIV vaccination between African and non-African vaccine recipients may have potentially contributed to the failure of multiple recent phase 2b/3 vaccine trials in African populations. We herein investigated the influence of race, geographic origin and sex in HVTN 204 vaccinees who received a VRC-HIV-DNA prime plus rAd5-HIV boost vaccine regimen on the Env-specific humoral immune response. An overall high frequency of vaccine-induced subtype C CN54rgp140 IgG1 and IgG3 responses were detected four weeks after final vaccination, with comparable titers of anti-CN54rgp140 and anti-IDR3_V3 IgG antibodies when results were stratified by race (Black Africans *vs* Caucasian Americans) or by geographic location (South Africa *vs* US). However, female sex was associated with increased systemic anti-CN54rgp140 IgG1, as well as a higher magnitude of IDR2_V2-specific IgG recognition compared to males.

These data are consistent with a recent meta-analysis of multiple HIV-1 vaccine regimens which reported that female sex is a strong predictor for a high HIV Env IgG responder status (20). Further, females are known to have 40% less HIV plasma viremia than males (21). This is relevant as increased vaccine-induced SIV-specific IgG isotypes levels have been linked with partial protection from SIV acquisition and reduced peak viremia in female macaques in pre-clinical HIV/SIV models (22). We also frequently observed no or very low levels of serum anti-CN54rgp140 IgA responses, especially in vaccinated females compared to males. In the RV144 clinical trial, reduced systemic levels of IgA correlated with reduced risk for HIV-1 acquisition (23). It was hypothesized that IgA antibodies can compete with IgG for HIV-1 Envelope antibody binding sites, thereby reducing antibody-mediated effector functions such as antibody-dependent cellular cytotoxicity (15). Our data therefore do not support the hypothesis that differences in vaccine-induced humoral immune responses between racial or geographic groups contributed to the failure of the HVTN 702 and 705 HIV vaccine efficacy trials, which were conducted primarily among young African women at high risk of HIV infection (6, 24).

Higher levels of vaccine- and infection-induced immunity in females have also been previously reported (5, 25–27). For example, females generally mount a higher strain-specific antibody responses to influenza vaccine and a better vaccine efficacy (as measured by hospitalization and mortality rate) than males (27–29). Here we extend these findings to more novel vaccine approaches using naked DNA prime and an Adenoviral vector boost. Differences in sex hormones and the fact that various immune-related genes are X-linked with greater activation in females than males due to females possessing two X-chromosomes may contribute to the differences observed in our study (27, 30, 31). Nonetheless, it is important to note that factors other than female sex do play a significant role in infection dynamics. The South African HVTN 702 trial reported over a 3-fold increased cumulative risk for HIV acquisition in women when compared to men despite mounting high titers of functional anti-gp120 and anti-gp140 antibodies. The increased risk was likely attributed to increased genital inflammation resulting from sexually transmitted infections coupled with socioeconomic vulnerabilities that expose women to an increased “risky” lifestyle and a greater force of HIV infection compared to men (6). Our finding of elevated pre-vaccination anti-CN54rgp140-specific IgG1 titers, particularly in female vaccinees from South Africa has also been previously reported by others (32–35) and may stem from antibody cross-reactivity resulting from exposure to environmental antigens such as the intestinal microbiome or others (36).

People of Black African origin living with HIV have been reported to have higher HIV-specific IgG levels than Caucasian Americans with HIV (37). However, only a few clinical trials have systematically compared differences in the induction of HIV-specific antibodies between Black Africans and Caucasians during HIV clinical trials (4, 38–40). We have recently reported similar vaccine-induced IgG and IgA titers against consensus B Envelope antigens/HIV antigens from non-clade C isolates amongst South Africans, African Americans, and Caucasians from the US who participated in the HVTN 204 trial (17). Here, we extend these findings and show comparable titers of anti-CN54rgp140 binding antibodies isotypes between Black people from any of the studied geographic regions and Caucasians from the US following immunization with the same vaccine regimen.

The epitope specificity of anti-HIV-1 Env antibodies elicited in HVTN 204 trial participants aligns with previous observations for this vaccine regimen (19). RV172 trial participants in East Africa, receiving the same VRC-HIVDNA and rAd5-HIV vaccine combination as HVTN 204 participants (41), recognized the same Env regions in C1, V3, and gp41 (19). IDR2_V2 responses, however, were largely absent in RV172 participants studied in Horvath et al. (19). Intriguingly, IgG responses targeting the linear IDR2_V2 epitope (HXB2 163-177) were previously detected only following immunization regimens including clade E- or AE-based immunogens (14, 19, 42–44), whereas the HVTN 204 vaccine regimen is composed of Env glycoproteins from clades A, B and C (8). Unfortunately, demographic data for the participants studied during previous peptide array analyses were unavailable, precluding commentary on sex biases in Env responses within the analyzed trials (14, 19, 42, 45–48). Nevertheless, in contrast to the more frequent IDR2_V2 responses in females observed for HVTN 204, V2 responses were highly frequent in the all-male participants of the Vax003 trial, which tested a monomeric gp120 immunogen (14). Additionally, in RV144, no correlation was found between immune responses associated with protection and sex (13), highlighting the need for further exploration of sex and race disparities in vaccine-induced antibody epitope specificity and to disentangle individual effects of demographic variables.

Limitations of this study include a small sample size due to the unavailability of a third of the targeted samples from the HVTN 204 trial at the time of analysis. While the sample size is limited, we identified the HVTN 204 trial as best suited for our purpose, as phase 2 vaccine trials are typically conducted only on a single continent. Peptide array analysis only focused on Black Africans and US Caucasians because of limited availability of custom peptide arrays at the time. Moreover, this assay only detects antibodies to linear, non-glycosylated epitopes but not conformational ones. A greater sample size would enable a more powerful statistical multivariate analysis. Future studies should therefore optimally explore the effects of race, geography and sex on vaccine-induced neutralizing antibody and cell-mediated immune responses in larger cohorts. Nonetheless, despite the small sample size, we were able to show a significant influence of sex on vaccine-induced IgG Env-specific binding antibody titers and recognition of certain Env regions in HVTN 204, that associated with protection during the RV144 trial (13, 14). Hence, these findings shed more light on our understanding of the influence of sex in determining the immunogenicity of DNA and/or Adenoviral vector vaccine approaches for HIV and potentially extending to other pathogens.

## Conclusion

In conclusion, female sex was most associated with differences in the vaccine-induced HIV-Env-specific antibody response, while these differences were less prominent when stratifying assay results for HVTN 204 participants by geographic location or race. Thus, biological factors driving these sex-based differences should be further examined in other HIV vaccine-induced humoral and cell-mediated responses.

## Data Availability

The raw data supporting the conclusions of this article will be made available by the authors, without undue reservation.

## Supplementary material

Supplementary Figure 1 HVTN 204 trial participants from the vaccine arm with respect to country (United States of America vs South Africa) and race (Black vs Caucasian) who participated in this study.

Supplementary Figure 2 South African females have increased anti-CN54rgp140-specific IgG1 background signal. Optical densities (OD) in participants with background signal pre-vaccination **(A)** IgG1 (n=37), **(B)** IgG3 (n=73) and **(C)** IgA (n=90) stratified by sex, race and geographical region. Each dot represents one individual; round dots indicate males and triangles represent females. Median lines are indicated in graphs. Statistical analysis was performed using the Mann-Whitney U-test.

Supplementary Figure 3 Magnitude of anti-CN54rgp140-specific IgG1, IgG3 and IgA titers stratified by geographic location, sex and race of vaccinated participants. Antibody titers in vaccinated participants for **(A)** IgG1 (n = 115), **(B)** IgG3 (n = 134) and **(C)** IgA (n = 105) stratified by sex, race and geographical region. Each dot represents one individual; round dots indicate males and triangles represent females. Median lines are indicated in graphs. Statistical analysis was performed using the Mann-Whitney U-test.

Supplementary Figure 4 HIV-1 Env recognition of HVTN 204 participants shows four immunodominant epitopes. IgG epitope recognition along the HIV-1 Env protein for all participant groups. The frequency of responders (FOR; upper panel) and the mean fluorescence intensities (mean FI; lower panel) plotted against individual antigenic regions along the entire HIV-1 Env as included in the 10 full-length Env immunogen sequences comprising the array backbone. Each row of the respective heat maps displays the Env-specific IgG responses of HVTN 204 participants stratified by geographic location and sex, tested four weeks after the last vaccination. IgG responses against individual antigenic regions were considered positive if the corresponding FI was above 3,500 after subtraction of the pre-vaccination value. The mean FI was calculated using the maximum FI values per position for each participant, only if peptide-specific IgG responses occurred in at least 25% of the vaccinees. IDRs 1–4 are indicated by red lines.
