# Supplementary figures and images for: Effects of sex but not race and geographic origin on vaccine-induced HIV-specific antibody responses
Source: Front Immunol. 2025 Sep 23;16:1601865. doi: 10.3389/fimmu.2025.1601865 (PMC12500573; doi:10.3389/fimmu.2025.1601865)

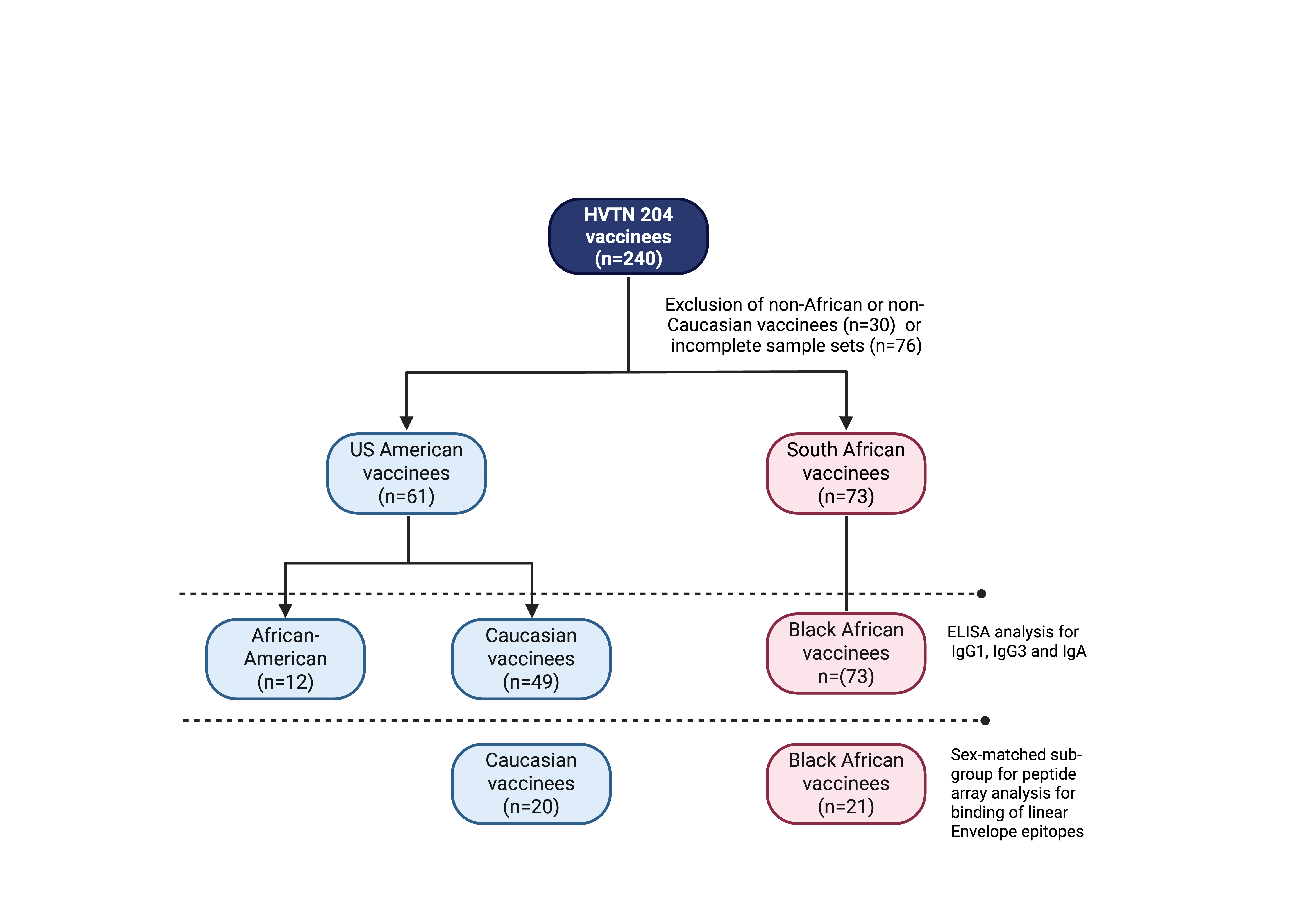

Supplement: Supplementary Figure 1 — HVTN 204 trial participants from the vaccine arm with respect to country (United States of America vs South Africa) and race (Black vs Caucasian) who participated in this study. [file Image1.jpeg]

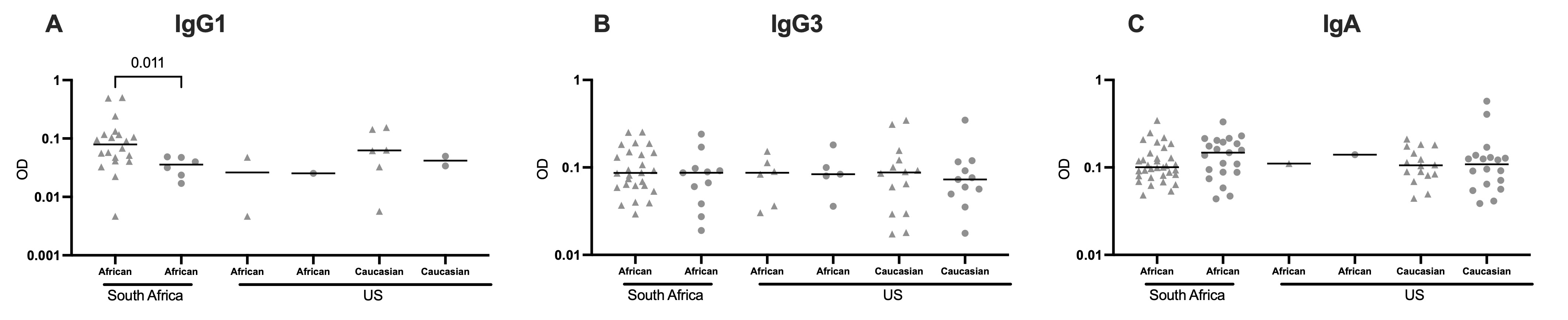

Supplement: Supplementary Figure 2 — South African females have increased anti-CN54rgp140-specific IgG1 background signal. Optical densities (OD) in participants with background signal pre-vaccination (A) IgG1 (n=37), (B) IgG3 (n=73) and (C) IgA (n=90) stratified by sex, race and geographical region. Each dot represents one individual; round dots indicate males and triangles represent females. Median lines are indicated in graphs. Statistical analysis was performed using the Mann-Whitney U-test. [file Image2.jpeg]

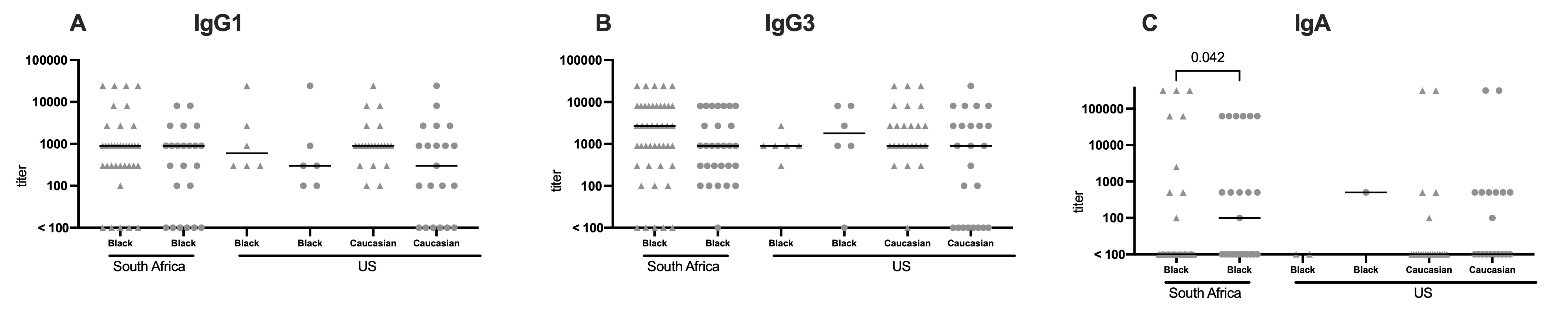

Supplement: Supplementary Figure 3 — Magnitude of anti-CN54rgp140-specific IgG1, IgG3 and IgA titers stratified by geographic location, sex and race of vaccinated participants. Antibody titers in vaccinated participants for (A) IgG1 (n = 115), (B) IgG3 (n = 134) and (C) IgA (n = 105) stratified by sex, race and geographical region. Each dot represents one individual; round dots indicate males and triangles represent females. Median lines are indicated in graphs. Statistical analysis was performed using the Mann-Whitney U-test. [file Image3.jpeg]
